# Supplementary material for: Effect of Novel AKT Inhibitor Vevorisertib as Single Agent and in Combination with Sorafenib on Hepatocellular Carcinoma in a Cirrhotic Rat Model
Source: Int J Mol Sci. 2022 Dec 19;23(24):16206. doi: 10.3390/ijms232416206 (PMC9784348; doi:10.3390/ijms232416206)
Supplement: Supplementary file 1 [file ijms-23-16206-s001.zip › ijms-2057287-supplementary.pdf]

Supplementary information:

Effect of novel AKT inhibitor Vevorisertib as single agent and in combination with Sorafenib on hepatocellular carcinoma in a cirrhotic rat model

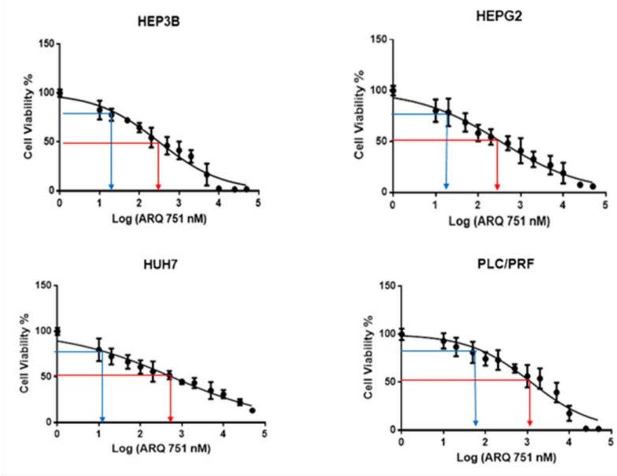

Supplementary Figure S1: Cell viability assay on cell lines. MTT assay was used to determine viability of Hep3B, HepG2, HuH7, PLC/PRF cells treated with different concentrations of Sorafenib or ARQ 751(vevorisertib) incubated during 48 hours. All experiments were done in triplicates and repeated three times. IC20 blue line, IC50 red line. Data are presented as mean ± SD.

		HEP3B	HUH7	HEPG2	PLC/PRF/5
Vevorisertib	IC_{20}	0.02±0.002	0.01±0.001	0.003±0.0003	0.04±0.009
	IC_{50}	0.32±0.05	0.58±0.05	0.34±0.02	1.24±0.07
Sorafenib	IC_{20}	0.89±0.03	0.90±0.01	1.48±0.05	1.09±0.06
	IC_{50}	6.68±0.03	11.58±0.03	10.53±0.04	12.69±0.04
IC_{50} Potency ratio	Sorafenib IC_{50}/ Vevorisertib IC_{50}	21	20	30	10

Supplementary Table S1: IC20 and IC50 of Vevorisertib, Sorafenib and the IC50 potency ratio. Inhibitory concentrations IC20 and IC50 for Sorafenib and Vevorisertib, using Hep3B, HuH7, HepG2, PLC/PRF cell lines. Data are presented as mean ± SD. IC50 potency ratio between Sorafenib and Vevorisertib was calculated for Hep3B, HuH7, HepG2, PLC/PRF cell lines as Sorafenib IC_{50}/ Vevorisertib IC_{50}.

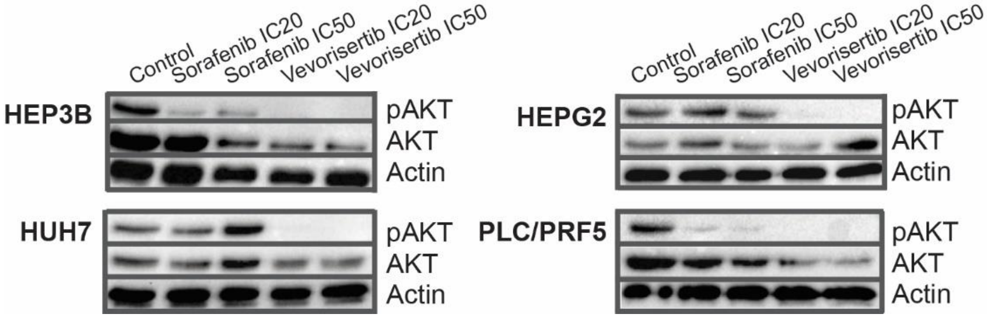

Supplementary Figure S2: Effect of Vevorisertib treatment on phosphorylation of AKT. Representative western blot analysis of pAKT, AKT and Actin using Hep3B, HuH7, HepG2, PLC/PRF cell lines.

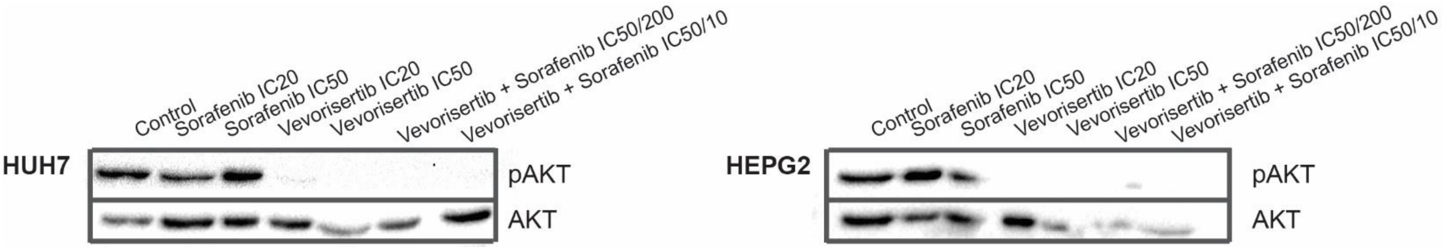

Supplementary Figure S3: Effect of Vevorisertib and Vevorisertib + Sorafenib treatment on phosphorylation of AKT. Representative western blot analysis of pAKT and AKT using HuH7 and HepG2 cell lines.
